# Erenumab Impact on Sleep Assessed With Questionnaires and Home-Polysomnography in Patients With Migraine: The ERESON Study

**DOI:** 10.3389/fneur.2022.869677

**Published:** 2022-05-13

**Authors:** Gaia Pellitteri, Sara Pez, Annacarmen Nilo, Andrea Surcinelli, Gian Luigi Gigli, Christian Lettieri, Mariarosaria Valente

**Affiliations:** ^1^Clinical Neurology Unit, Santa Maria della Misericordia University Hospital, Udine, Italy; ^2^Department of Medical Area (DAME), University of Udine, Udine, Italy; ^3^Neurology Unit, Santa Maria della Misericordia University Hospital, Udine, Italy

**Keywords:** migraine, PACAP, CGRP, erenumab, PSQI, sleep efficiency, polysomnography

## Abstract

**Introduction:**

Migraine and sleep share a complex and unclear relationship. Poor sleep may trigger migraine attacks; migraine, in turn, is frequently associated with sleep disorders. Few previous studies used questionnaires to assess sleep changes in patients who were treated with migraine-preventive medications (MPMs). More extensive polysomnography (PSG)-based studies for this purpose were not available.

**Objective:**

To investigate possible sleep changes in patients with migraine treated with erenumab, using validated sleep questionnaires and home-PSG.

**Methods:**

This observational, prospective, open-label pilot study was conducted at the Clinical Neurology Unit Headache Center of Udine University Hospital from 2020 to 2021. Patients were treated with erenumab as monotherapy or add-on treatment for migraine prevention. Sleep changes were evaluated with questionnaires and polysomnographic recordings at baseline, after 3 and 12 months of treatment. Erenumab efficacy and safety in migraine prophylaxis were also investigated.

**Results:**

Twenty-nine patients completed 3 months of follow-up, whereas 15 patients completed 12 months. We found a weak trend of improvement in daytime somnolence after 3 months of treatment, with stronger results after 12 months (median Epworth Sleepiness Scale (ESS) score from 6.0 to 4.0, *p* = 0.015); a significant improvement in subjective sleep quality (median Pittsburgh Sleep Quality Index (PSQI) total score from 7 to 5; *p* = 0.001) was also observed. Home-PSG showed a significant increase in objective sleep efficiency (SE), both after 3 (from 88.1 to 91.0, *p* = 0.006) and 12 months (from 87.1 to 91.0, *p* = 0.006) of treatment. In addition, our data confirmed erenumab effectiveness and safety in migraine prevention.

**Conclusion:**

Our study demonstrated an improvement in both subjective and objective sleep quality in patients treated with a migraine-preventive therapy. Erenumab, in particular, does not cross the blood-brain barrier (BBB), thus a direct effect on sleep is unlikely. Future studies are needed to better understand the mutual influence between migraine and sleep disorders.

## Introduction

Migraine is a common disabling primary headache disorder with high socio-economic burden and personal impact. It typically occurs with recurrent attacks of pulsating quality and unilateral location, associated with autonomic symptoms, such as nausea, photophobia, and phonophobia, as defined by the International Classification of Headache Disorders, 3rd edition (ICHD-III) (1). Migraine etiology and pathophysiological mechanisms are complex and not fully understood. It is well-known that various triggers may precipitate migraine attacks, such as changes in lifestyle, physical activity, diet, and hormones and sleep disturbances (1, 2).

Increasing evidence suggests that sleep and migraine share a multifaceted and bidirectional relationship. Patients with migraine frequently report poor sleep quality and excessive daytime sleepiness (EDS). On the other hand, sleep disruption can be a trigger of migraine attacks, which often benefit of sufficient restful sleep (3–6). In clinical settings, physicians ordinarily consider sleep as one aspect of migraine treatment, selecting medications with sedative side effects, in an attempt to improve sleep as a protective factor against migraine attacks.

Several polysomnographic studies identified a decreased sleep efficiency (SE) and alterations in sleep architecture in patients with migraine when compared to healthy subjects (7–9). Furthermore, among adult patients with migraine, a high prevalence of sleep disorders has been found and can range from insomnia to more specific disorders, such as Restless Legs Syndrome (RLS) and parasomnias (10–15). Together, these findings suggest to consider migraine and sleep problems as conditions with common pathophysiologic elements that still are partly obscure. Few previous studies evaluated sleep changes in patients treated with migraine-preventive treatments by means of questionnaires (16–19). For example, a recent study investigated possible sleep changes in patients with chronic migraine effectively treated with a greater occipital nerve block, showing a significant improvement in scores of different clinical questionnaires evaluating sleep after 3 months of treatment (19). However, more extensive clinical and polysomnographic trials for this purpose are not currently available.

In the last years, the development of new monoclonal antibodies toward calcitonin gene-related peptide (CGRP) or its receptor has opened an innovative therapeutic scenario. Erenumab, in particular, was the first anti-CGRP receptor antibody employed in the clinical practice for migraine prevention (20).

The aim of the present study was to investigate possible sleep changes in patients with episodic and chronic migraine treated with erenumab, assessed with validated sleep questionnaires and polysomnography (PSG) performed at baseline, after 3 and 12 months of treatment.

## Methods

### Study Design, Eligibility Criteria, and Ethical Approval

This observational, prospective, open-label pilot study was performed at the Clinical Neurology Unit Headache Center of Udine University Hospital (Udine, Italy) between 2020 and 2021. It included patients treated with erenumab for the first time, prescribed on clinical indication as monotherapy or add-on treatment to other migraine-preventive medications (MPMs). Enrolled patients fulfilled all the established inclusion criteria and none of the exclusion criteria is reported in Table 1. This study was evaluated and approved by the Regional Ethics Committee of Friuli Venezia Giulia, Italy (approval code: CEUR-2020-OS-230) and was conducted in accordance with the Declaration of Helsinki code of ethics and the Good Clinical Practice guidelines. All patients included in the study provided written informed consent. The authors confirm full access to all anonymized study data.

**Table 1:** Study eligibility criteria.

	***Comments***
**Inclusion criteria**	
Clinical diagnosis of chronic or episodic migraine with ≥4 MMD	*According to the International Classification of Headache Disorders, 3^*rd*^ edition (ICHD-III) (1)*
Failure of ≥2 previous MPMs	*Due to lack of response (at the therapeutic dose for ≥ 6 weeks of treatment), intolerance or contraindication*
Steadiness in type and dosage of possible concurrent MPMs	*For ≥ 2 months before the enrollment*
A complete headache diary	*For ≥ 2 months before the enrollment*
**Exclusion criteria**	
TTH, TACs, other primary (except migraine) or secondary headache diagnoses	*Patients with MOH and pre-existing migraine were included*
Clinical history of sleep disorders (insomnia, sleep-related breathing disorders, central hypersomnolence, CRSD, parasomnias, sleep-related movement disorders)	*According to the International Classification of Sleep Disorders, 3^*rd*^ edition (ICSD-3) (21); only migraine-related insomnia was not considered as exclusion criteria*
Current treatment with BoNT-A as migraine prophylaxis	*For ≥ 4 months before the enrollment*
Ongoing therapies with hypnotic, stimulant or psychoactive medications	*Except drugs used as migraine prophylaxis (e.g. amitriptyline, flunarizine)*
Substance abuse, excessive intake of alcohol, caffeine or other stimulant products	*E.g., ginseng, taurine, guarana*
Major medical complaints or psychiatric disorders which may influence sleep quality	*Due to their influence on sleep quality*
Body mass index >30 or <18 kg/m^2^	*As predisposing conditions to sleep disorders*
Age > 65	*To avoid sleep disorders related to the elderly*
Contraindications to the treatment with erenumab and conditions excluded from pivotal studies	*i.e., hypersensitivity to medication components, childhood, pregnancy, breastfeeding, major CVD*

*MMD, monthly migraine days; MPM, migraine-preventive medication; TTH, tension type headache; TACs, trigeminal autonomic cephalalgias; MOH, medication overuse headache; CRSD, circadian rhythm sleep-wake disorders; BoNT-A, botulinum neurotoxin type A; CVD, cardiovascular diseases*.

### Data Collection

#### Interview and Clinical Examination

The following socio-demographic characteristics and relevant clinical data were collected: age, gender, body mass index, major comorbidities, current medications, age at onset of migraine, family history of migraine, number, and type of previous and ongoing MPMs. Enrolled patients underwent neurological examinations that were performed by an expert team of specialists in headache disorders, at the baseline visit (T0) and during the follow-up after 3 (T1) and 12 months (T2) of treatment with erenumab. At each visit, detailed clinical characteristics of migraine were collected, such as frequency and intensity of attacks, pain quality and localization, presence of aura, associated vegetative symptoms, migraine triggers, and classes and number per month of symptomatic treatments used. We also evaluated, at T1 and T2 follow-up visits, how many patients reported a subjective improvement in attack duration and response to symptomatic medications after taking erenumab, measured as a dichotomous variable (yes/no). In addition, we collected data about any side effects reported during the treatment.

#### Headache Diary and Questionnaires

Patients were requested to fill out a headache diary for 2 months before the enrollment and throughout the entire study period to evaluate changes in frequency and severity of migraine attacks during the treatment. Migraine frequency was expressed as monthly migraine days (MMDs), while the severity of pain was a 3-point score (1 = mild, 2 = moderate, and 3 = severe). For each patient, migraine frequency at baseline was defined as the mean of MMDs during the 2 months preceding the enrollment. After 3 and 12 months of treatment, it was defined as the mean of MMDs in the month preceding T1 and in the 2 months preceding T2, respectively. Patients were considered as responders if they reported ≥50% reduction in migraine frequency.

All patients also completed a series of questionnaires collected *via* face-to-face interviews at baseline, T1, and T2 follow-up visits. We used the Pittsburgh Sleep Quality Index (PSQI) and the Epworth Sleepiness Scale (ESS) (22, 23), aimed at exploring subjective sleep quality and daytime sleepiness, respectively. Disability and impact on daily living associated with migraine were evaluated using both the Migraine Impact and Disability Assessment Scale (MIDAS) and the Headache Impact Test, 6th edition (HIT-6) (24, 25). Additionally, we included the Beck Depression Inventory-II questionnaire (BDI-II) and the EuroQoL Visual Analogue Scale (EQ-VAS) in order to rate depressive symptoms and subjective quality of life, respectively (26, 27).

#### Home-PSG

Enrolled patients underwent three home-PSG recording sessions at times T0, T1, and T2. All recordings were performed for two subsequent nights. For our comparisons, we used all second-night recordings in order to limit the possible “first-night effect,” due to the discomfort caused by electrodes and other measurement devices (28, 29). Sleep recordings were performed with a battery-powered self-applicable device positioned on the forehead (Sleep Profiler^TM^, Advanced Brain Monitoring, Carlsbad, CA, USA). PSG recordings were uploaded to the Sleep Profiler^TM^ software and were initially analyzed by the automated scoring system. Finally, two different sleep specialists visually inspected each analysis, minimally editing as appropriate, to confirm staging accuracy. Further detailed information on Sleep Profiler^TM^ hardware and software, automated scoring system, and reliability of results in comparison with laboratory-PSG has been previously published (see Supplementary Materials) (30–32).

### Erenumab Treatment

All included patients met the eligibility criteria established by the European Headache Federation and the American Headache Society for the treatment with new monoclonal antibodies acting on the CGRP pathway (33, 34). Patients with migraine and coexistent medication overuse headache (MOH) were not detoxified prior to start erenumab treatment. All subjects received the first dose of erenumab 70 mg administered subcutaneously after completing a baseline PSG recording. All patients continued the treatment at a dose of 70 mg monthly for the first 3 months of the study, followed by a second home-PSG. During that period, any other ongoing MPM was not discontinued or modified. From the fourth month, a dose switch to 140 mg monthly was proposed according to treatment effectiveness and tolerability; modifying or withdrawing concurrent oral MPMs was also allowed according to the physician's judgment. The concomitant inoculation of botulinum neurotoxin type A (BoNT-A) was not permitted in any case. All patients received a monthly treatment with erenumab for 52 weeks, then completed the study with the third home-PSG.

### Study Endpoints

We defined a primary composite endpoint, such as variations in SE (I) derived from PSG (SE, %), in subjective sleep quality (II) measured by the PSQI score, and in EDS (III) assessed with the ESS score. SE ≥ 85%, PSQI score ≤ 5, and ESS score ≤ 10 were considered as normal thresholds (22, 23, 35). We compared all parameters collected at times T0, T1, and T2.

As secondary endpoints, we established the following issues: (i) changes in other PSG parameters, such as total sleep time (TST), duration of wake (W) and N1, N2, N3, and Rapid Eye Movement (REM) sleep stages, wake after sleep onset (WASO), sleep latency (SL), REM latency (RL), arousal index (number of arousal per hour, Ar/h), and awakening index (number of awakenings per hour, Aw/h); (ii) effects on migraine-related disability, measured by MIDAS and HIT-6 scales (24, 25); (iii) effects on mood, tested with the BDI-II questionnaire (26); (iv) subjective changes in life quality, assessed with the EQ-VAS (27). Finally, as erenumab has been recently approved and is still subject to additional monitoring by the regulatory agencies, we evaluated treatment effectiveness (v) and tolerability (vi), by using information from clinical interviews, headache diaries, and collecting patient-reported adverse events (AEs).

### Statistical Analysis

All data were collected in an *ad hoc* created database (Excel 2019, Microsoft Corp., Redmond, WA, USA). Data cleaning was performed before the data analysis. Continuous variables were summarized by descriptive statistics, expressed as median, interquartile interval, and range, and categorical variables by absolute frequencies and percentages. Migraine parameters, sleep parameters, and sleep scores were compared using Fisher's and McNemar's tests for categorical data and Wilcoxon signed-rank test for continuous data, with the threshold of significance of *p* < 0.05. All statistical analyses were performed using SPSS version 25.0 (IBM Corporation, Chicago, IL, USA).

## Results

### Patient Characteristics

A total of 29 patients met the study inclusion criteria and completed the follow-up after 3 months of treatment. Fourteen patients were lost at follow-up because of limited access to hospital services due to the Coronavirus Disease-19 (COVID-19) pandemic, whereas 15 patients (51.7%) completed a 12-month follow-up visit. For this reason, statistical analyses were conducted separately for data collected at 3 months on the whole sample (*n* = 29) and 3 and 12 months on a subgroup of patients (*n* = 15).

Patients included were predominantly women (86.2%), with a median age of 46.1 years (range 19–61) at baseline. All patients presented a long clinical history of the disease, mostly with chronic migraine (65.5%), refractory to different classes of MPMs (median 4.0, range 2–10). Thirteen patients (44.8%) with chronic migraine had also failed previous treatment with BoNT-A injection. In 17 of 29 patients (58.6%), erenumab was prescribed in an add-on to other ongoing oral MPMs. The main baseline demographic and clinical characteristics of patients are reported in detail in Table 2.

**Table 2:** Main baseline demographic and clinical characteristics of patients (*n* = 29).

**Gender, *n* (%)**		**Comorbidity, *n* (%)**	
Female	25 (86.2)	Depression	4 (13.8)
Male	4 (13.8)	Arterial hypertension	2 (6.9)
**Age (years)**		Dyslipidemia	1 (3.4)
Median (IQR)	46.1 (18)	Smoke	1 (3.4)
Range	19–61	Lupus erythematosus	1 (3.4)
**Body Mass Index**		Asthma	1 (3.4)
Median (IQR)	22.0 (4)	Fibromyalgia	1 (3.4)
Range	18–26	Autoimmune thyroiditis	1 (3.4)
**Type of migraine, *n* (%)**		**Family history of migraine, *n* (%)**	
Episodic migraine	10 (34.5)	Positive	9 (31.0)
Chronic migraine	19 (65.5)	**Age at migraine onset, years**	
Migraine with aura	3 (10.3)	Median (IQR)	20.0 (11)
Migraine without aura	26 (89.7)	Range	10–35
**Patients under MPM, *n* (%)**	17 (58.6)	**Previous failures with MPMs, *n***	
**MPM type, patient *n* (%)**		Median (IQR)	4.0 (4)
Venlafaxine	5 (17.2)	Range	2–10
Amitriptyline	4 (13.8)	**MPM classes, patient *n* (%)**	
Topiramate	3 (10.3)	Antidepressants	26 (89.7)
Lamotrigine	2 (6.9)	Antiepileptics	25 (86.2)
Flunarizine	1 (3.4)	Beta-blockers	17 (58.6)
Citalopram	1 (3.4)	Calcium antagonists	16 (55.2)
Propranolol	1 (3.4)	BoNT-A	13 (44.8)
***Patients with MOH, *n* (%)***	3 (10.3)	**Symptomatic drug intake, *n*/month**	
		Median (IQR)	8.0 (3)
		Range	3–16

*n, number; IQR, interquartile range; MPM, migraine-preventive medication; MOH, medication overuse headache; BoNT-A, botulinum neurotoxin A*.

### Migraine Severity, Disability, Mood Level, and Life Quality

Considering the entire study sample (*n* = 29), eight patients (27.6%) showed a significant ≥50% reduction of MMD (*p* = 0.001) and were considered responders at 3 months of follow-up. At baseline, 27.6% of patients reported the subjective severity of migraine attack as “severe,” while 69.0% of them as “moderate.” After 3 months of treatment, we found a significant improvement in subjective attack severity; in fact, only 17.2% of patients described the migraine attack as “severe,” whereas patients with “mild” attacks were increased from 3.4% up to 37.9%. Moreover, 69.0% of the patients reported a subjective reduction in the duration of the single migraine attack, and 65.5% an improvement in response to symptomatic drugs (see Table 3).

**Table 3:** Erenumab effect on migraine after 3 months of treatment in enrolled patients (*n* = 29).

	**T0 (baseline)**	**T1 (3 months)**	
**HEADACHE DIARY AND CLINICAL INTERVIEW**			
**Monthly migraine days**			
Median (IQR)	13.0 (9.8)	8.0 (10.0)	*p =* 0.001^*^
≥50% reduction, *n* (%)		8 (27.6)	
**Attack severity**, ***n*** **(%)**
Mild	1 (3.4)	11 (37.9)	*p =* 0.025^*^
Moderate	20 (69.0)	13 (44.8)	
Severe	8 (27.6)	5 (17.2)	
**Subjective reduction in migraine duration**, ***n*** **(%)**		20 (69.0)	
**Improved response to symptomatic drugs**, ***n*** **(%)**		19 (65.5)	
**CLINICAL QUESTIONNAIRES**			
**MIDAS score**			
Median (IQR)	45.0 (81.0)	24.0 (41.0)	*p =* 0.001^*^
≥50% reduction, *n* (%)		12 (41.4)	
**HIT-6 score**			
Median (IQR)	66.0 (9.0)	63.0 (36.0)	*p =* 0.001^*^
**BDI-II score**			
Median (IQR)	5.0 (6.0)	4.0 (7.0)	*p =* 0.01^*^
**EQ-VAS score**			
Median (IQR)	60.0 (33.0)	75.0 (15.0)	*p =* 0.001^*^

*IQR, interquartile range; n, number of patients; MIDAS, Migraine Impact and Disability Assessment Scale; HIT-6, Headache Impact Test, 6th edition; BDI-II, Beck Depression Inventory-II; EQ-VAS, EuroQoL Visual Analogue Scale*.

* *p < 0.05*.

The subgroup of patients at 12 months of follow-up (*n* = 15) showed a significant reduction from baseline in MMD and in subjective severity of migraine attacks (*p* = 0.001 and *p* = 0.002, respectively). Moreover, the proportion of patients reporting a reduction in duration of attacks was increased up to 73.3%. During the 12-month treatment period, 12 of 15 patients (78.5%) escalated the erenumab dose from 70 to 140 mg, and 11 (73.3%) withdrew the prior oral MPM because of erenumab effectiveness (see Table 4).

**Table 4:** Erenumab effect on migraine after 12 months of treatment (*n* = 15).

	**T0 (baseline)**	**T1 (3 months)**	**T2 (12 months)**	***T0–T2^†^***
**Headache diary and clinical interview**				
**Monthly migraine days**				
**Median (IQR)**	12.0 (9.0)	8.0 (11.0)	5.0 (8.0)	*p =* 0.001^*^
≥50% reduction, *n* (%)		4 (26.7)	6 (40.0)	
**Attack severity**, ***n*** **(%)**				
Mild	1 (6.7)	8 (53.3)	9 (60.0)	*p =* 0.002^*^
Moderate	10 (66.7)	5 (33.3)	6 (40.0)	
Severe	4 (26.7)	2 (13.3)	0 (0.0)	
**Subjective reduction in migraine duration**, ***n*** **(%)**			11 (73.3)	
**Improved response to symptomatic drugs**, ***n*** **(%)**			10 (66.7)	
**Erenumab dose escalation to 140 mg**, ***n*** **(%)**		12 (78.5)		
**CLINICAL QUESTIONNAIRES**				
**MIDAS score**				
Median (IQR)	45.0 (62.0)	25.0 (30.0)	15.0 (28.0)	*p =* 0.001^*^
≥50% reduction, *n* (%)		6 (40.0)	12 (80.0)	
**HIT-6 score**				
Median (IQR)	68.0 (7.0)	64.0 (7.0)	64.0 (7.0)	*p =* 0.001^*^
**BDI-II score**				
Median (IQR)	4.0 (8.0)	3.0 (6.0)	2.0 (6.0)	*p =* 0.003^*^
**EQ-VAS score**				
Median (IQR)	69.0 (30.0)	75.0 (15.0)	80.0 (13.0)	*p =* 0.002^*^

*IQR, interquartile range; n, number of patients; MIDAS, Migraine Impact and Disability Assessment Scale; HIT-6, Headache Impact Test, 6th edition; BDI-II, Beck Depression Inventory-II; EQ-VAS, EuroQoL Visual Analogue Scale*.

† *Referring to comparisons between T0 and T2 values*.

* *p < 0.05*.

In the whole sample (*n* = 29), the median MIDAS total score was 45.0 at baseline, which improved to 24.0 after 3 months of follow-up. A ≥50% reduction of MIDAS score was achieved by 41.4% of patients. Considering the subgroup of 15 patients, a clinically relevant improvement in MIDAS score was confirmed both at 3- and 12-month follow-up visits. HIT-6 score also improved significantly after 3 months, with a 3-point reduction in 29 patients (*p* = 0.0001). Among 15 patients, changes from baseline in HIT-6 scores were – 4.0 after 3 months (*p* = 0.001) and – 6.0 after 12 months (*p* = 0.001). Lastly, we found a significant change in the median of BDI and EQ-VAS scale scores (*p* = 0.003 and *p* = 0.001, respectively) at a 3-month follow-up on 29 patients. Among the subgroup of 15 patients, the median scores on BDI and EQ-VAS scale were confirmed significantly improved from baseline, both to 3 and 12 months of follow-up (see Tables 3, 4).

All patients showed an optimal compliance to the treatment. The most common AE was constipation, reported by six patients (20.7%); other AEs were observed in isolated cases, particularly asthenia (3.4%), irritability (3.4%), and Raynaud's phenomenon (3.4%). All AEs were reported as mild; none of the patients discontinued the treatment because of side effects.

### Subjective and Objective Sleep Quality Parameters

#### Sleep Questionnaires

**Figure 1:**
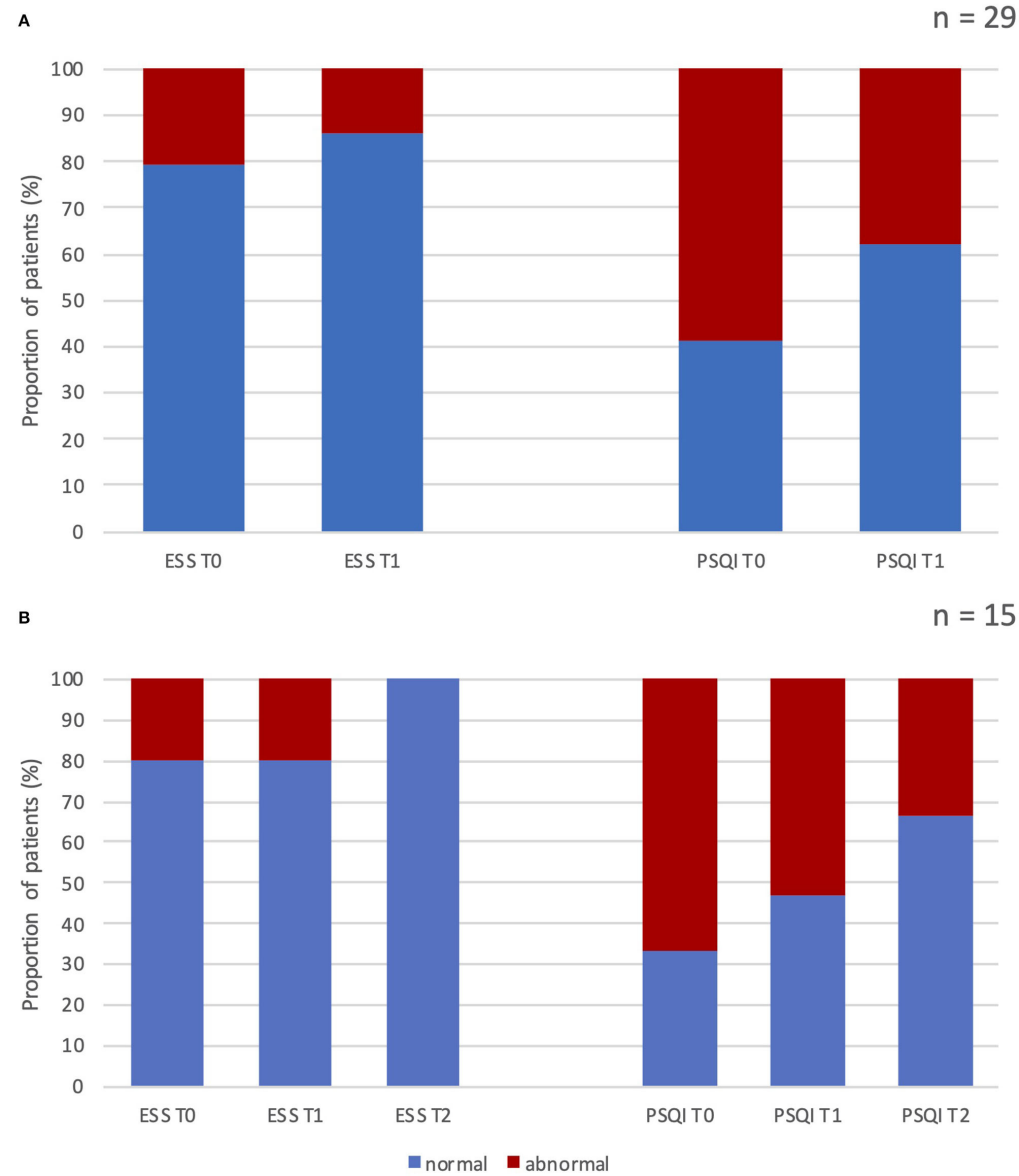
Percentage of patients with normal vs. abnormal scores at the Epworth Sleepiness Scale and Pittsburgh Sleep Quality Index before and after erenumab treatment. **(A)** Results after 3 months of treatment in all patients enrolled (*n* = 29). **(B)** Results after 3 and 12 months of treatment in patients who completed the 12-month follow-up (*n* = 15). ESS, Epworth Sleepiness Scale; PSQI, Pittsburgh Sleep Quality Index; T0, score at baseline; T1, score after 3 months of treatment; T2, score after 12 months of treatment; n, number of patients.

At baseline, six (20.7%) of all enrolled patients (*n* = 29) had pathological ESS scores (>10). After 3 months of treatment, we observed pathological scores in only three subjects. None of the patients with 1 year of follow-up (*n* = 15) showed abnormal ESS values (Figure 1). Median ESS values showed a weak trend of improvement in levels of daytime somnolence after 3 months of treatment, with stronger results in the sub-analysis on 15 patients at 12-month follow-up. In this subgroup, there was a significant reduction in the median ESS score (from 6.0 to 4.0, *p* = 0.015). The median baseline total PSQI score was 7.0 and 17 patients (58.6 %) had scores >6, indicating a global poor sleep quality. After 1 year, we found a significant change (from 7 to 5; *p* = 0.001) in the median PSQI total score (see Tables 5, 6).

**Table 5:** Results of sleep questionnaires and home-polysomnography recordings at baseline and after 3 months of treatment with erenumab (*n* = 29).

	**T0 (baseline)**	**T1 (3 months)**	
**Sleep questionnaires**			
ESS (total score)			
Median (IQR)	6.0 (7.0)	5.0 (7.0)	*p =* 0.094
PSQI (total score)			
Median (IQR)	7.0 (3.0)	5.0 (2.0)	*p =* 0.018^*^
**Home-PSG parameters**			
N1 duration (%)			
Median (IQR)	12.6 (5.4)	12.9 (5.2)	*p =* 0.552
N2 duration (%)			
Median (IQR)	46.2 (10.9)	46.3 (7.0)	*p =* 0.914
N3 duration (%)			
Median (IQR)	18.1 (10.1)	19.1 (6.8)	*p =* 0.496
REM duration (%)			
Median (IQR)	20.5 (4.3)	21.7 (6.1)	*p =* 0.880
W duration (%)			
Median (IQR)	11.9 (8.5)	9.0 (6.2)	*p =* 0.008^*^
TST (min)			
Median (IQR)	396.0 (78.0)	408.0 (102.0)	*p =* 0.225
WASO (min)			
Median (IQR)	33.0 (18.0)	23.0 (16.0)	*p =* 0.001^*^
SL (min)			
Median (IQR)	15.0 (17.0)	16.0 (25.5)	*p =* 0.811
RL (min)			
Median (IQR)	84.0 (87.5)	83.0 (54.0)	*p =* 0.417
Sleep efficiency (%)			
Median (IQR)	88.1 (8.5)	91.0 (6.2)	*p =* 0.006^*^
Arousal index			
Median (IQR)	17.4 (13.0)	17.6 (9.3)	*p =* 0.430
Awakening index			
Median (IQR)	4.0 (2.4)	3.5 (2.0)	*p =* 0.001^*^

*IQR, interquartile range; n, number of patients; ESS, Epworth Sleepiness Scale; PSQI, Pittsburgh Sleep Quality Index; PSG, polysomnography; N1, NREM1 sleep stage; N2, NREM2 sleep stage; N3, NREM3 sleep stage; REM, REM (Rapid Eye Movement) sleep stage; W, wake stage; TST, total sleep time; WASO, wake after sleep onset; SL, sleep latency; RL, REM latency; min, minutes*.

* *p < 0.05*.

**Table 6:** Results of sleep questionnaires and home-polysomnography recordings at baseline and after 12 months of treatment with erenumab (*n* = 15).

	**T0 (baseline)**	**T1 (3 months)**	**T2 (12 months)**	***T0–T2^†^***
**Sleep questionnaires**				
ESS (total score)				
Median (IQR)	6.0 (6.0)	5.0 (7.0)	4.0 (4.0)	*p =* 0.006^*^
PSQI (total score)				
Median (IQR)	7.0 (3.0)	6.0 (3.0)	5.0 (3.0)	*p =* 0.001^*^
**Home-PSG parameters**				
N1 duration (%)				
Median (IQR)	13.1 (6.5)	13.0 (5.8)	12.8 (6.9)	*p =* 0.754
N2 duration (%)				
Median (IQR)	44.8 (7.5)	46.3 (5.0)	42.0 (3.7)	*p =* 0.349
N3 duration (%)				
Median (IQR)	21.0 (10.4)	20.5 (6.4)	21.9 (7.7)	*p =* 0.256
REM duration (%)				
Median (IQR)	20.6 (4.1)	19.8 (6.6)	21.1 (5.2)	*p =* 0.712
Wake duration (%)				
Median (IQR)	12.9 (6.6)	11.3 (6.5)	9.0 (4.2)	*p =* 0.003^*^
TST (min)				
Median (IQR)	396.0 (60.0)	402.0 (120.0)	402.0 (96.0)	*p =* 0.293
WASO (min)				
Median (IQR)	37.0 (18.0)	23.0 (17.0)	24.0 (18.0)	*p =* 0.012^*^
SL (min)				
Median (IQR)	16.0 (19.0)	15.0 (34.0)	9.0 (10.0)	*p =* 0.014^*^
RL (min)				
Median (IQR)	78.0 (87.0)	69.0 (66.0)	91.0 (88.0)	*p =* 0.820
Sleep efficiency (%)				
Median (IQR)	87.1 (6.6)	88.7 (6.5)	91.0 (4.2)	*p =* 0.003^*^
Arousal index				
Median (IQR)	13.3 (12.7)	15.1 (7.8)	14.5 (10.8)	*p =* 0.820
Awakening index				
Median (IQR)	3.5 (2.0)	2.8 (2.0)	3.0 (1.8)	*p =* 0.094

*IQR, interquartile range; n, number of patients; ESS, Epworth Sleepiness Scale; PSQI, Pittsburgh Sleep Quality Index; PSG, polysomnography; N1, NREM1 sleep stage; N2, NREM2 sleep stage; N3, NREM3 sleep stage; REM, REM (Rapid Eye Movement) sleep stage; W, wake stage; TST, total sleep time; WASO, wake after sleep onset; SL, sleep latency; RL, REM latency; min, minutes*.

† *Referring to comparisons between T0 and T2 values*.

* *p < 0.05*.

Ten subjects (66.7%) had PSQI abnormal score at baseline, whereas the 53.3% and 33% of patients showed altered PSQI total score after 3 and 12 months of treatment, respectively (see Figure 1).

**Figure 2:**
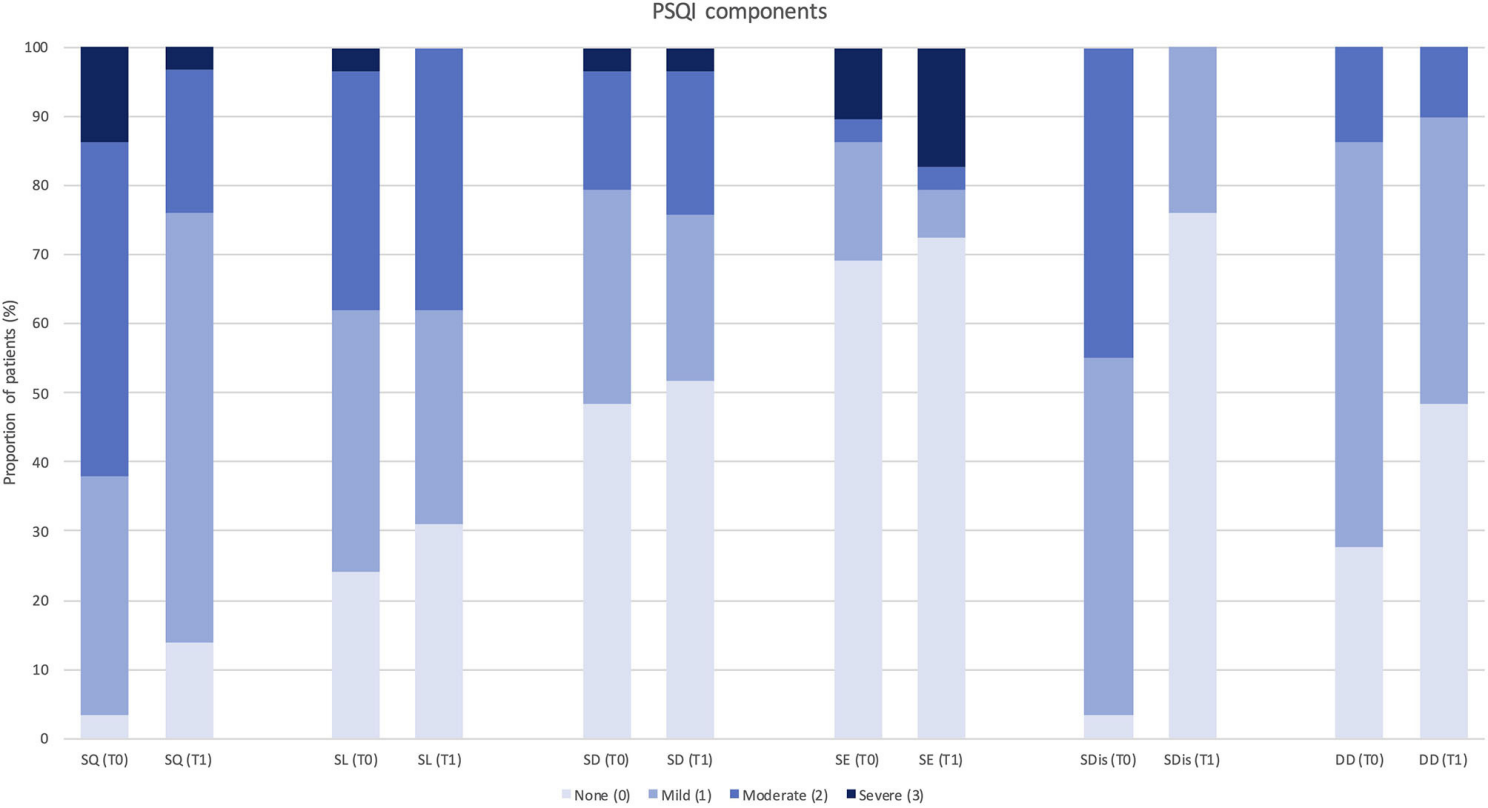
Change in Pittsburgh Sleep Quality Index component score after 3 months of treatment with erenumab (*n* = 29). PSQI, Pittsburgh Sleep Quality Index; SQ, subjective sleep quality; SL, sleep latency; SD, sleep duration; SE, habitual sleep efficiency; SDis, sleep disturbances; DD, daytime dysfunction; T0, score at baseline; T1, score after 3 months of treatment.

**Figure 3:**
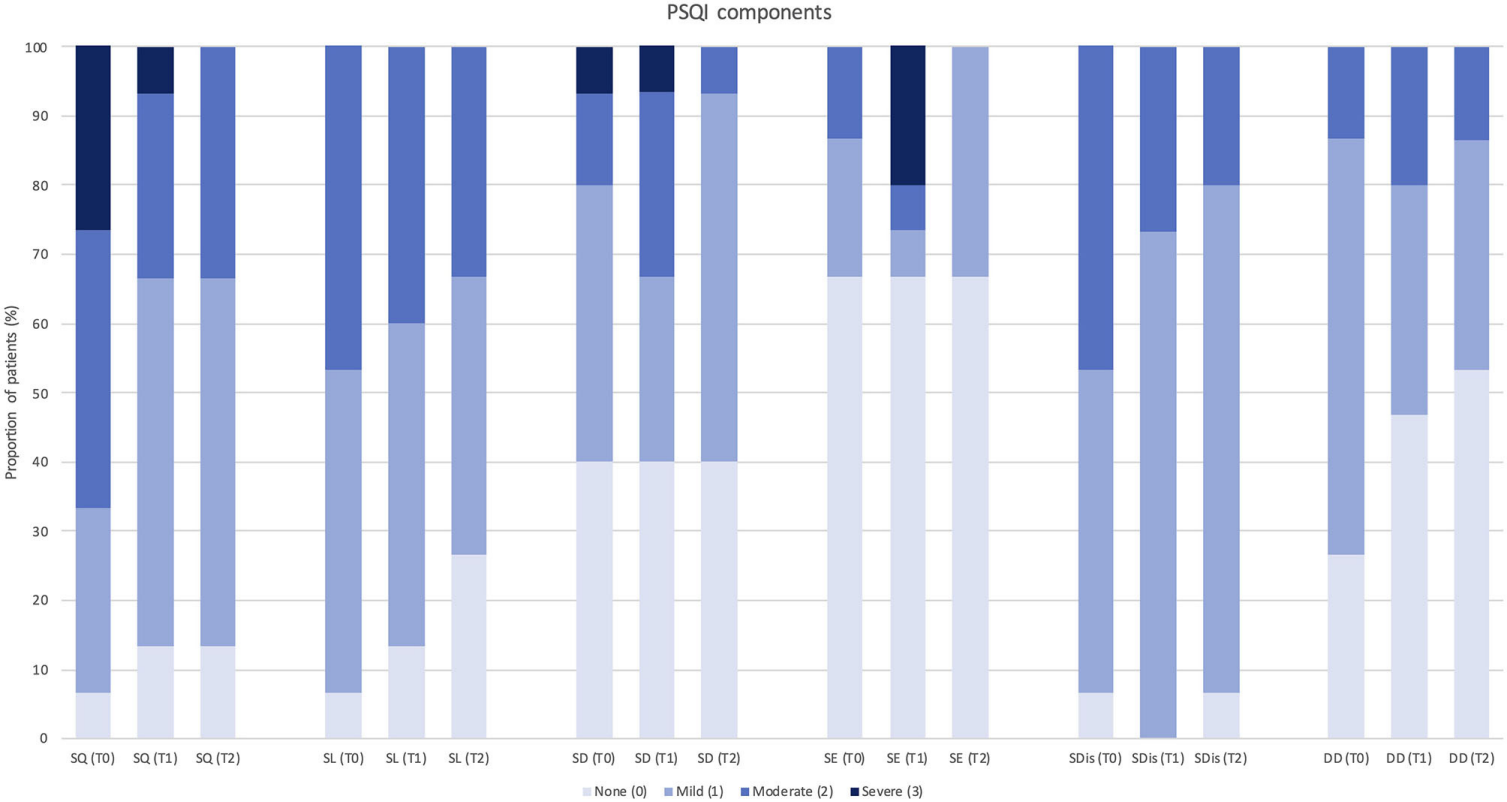
Change in each Pittsburgh Sleep Quality Index component score after 12 months of treatment with erenumab (*n* = 15). PSQI, Pittsburgh Sleep Quality Index; SQ, subjective sleep quality; SL, sleep latency; SD, sleep duration; SE, habitual sleep efficiency; SDis, sleep disturbances; DD, daytime dysfunction; T0, score at baseline; T1, score after 3 months of treatment; T2, score after 12 months of treatment.

Each PSQI component (such as subjective sleep quality, SL, sleep duration, habitual SE, sleep disturbances, and daytime dysfunction) was also analyzed separately comparing the distribution of scores (from a minimum of 0 = normal to a maximum of 3 = severe alteration) among patients at baseline, 3 months, and 12 months of follow-up (see Figures 2, 3).

Despite a global trend of improvement in single PSQI component scores, significant changes were observed in “subjective sleep quality” (*p* = 0.023) and “sleep disturbances” (*p* = 0.034) at 3-month follow-up (*n* = 29), and in “daytime dysfunction” (*p* = 0.046) at 12-month follow-up (*n* = 15). Only one PSQI component (i.e., use of sleeping medications) was not assessed, as none of the patients was using hypnotic drugs according to study exclusion criteria.

#### Home-PSG

Objective SE was significantly increased after erenumab treatment, both at 3-month (from 88.1 to 91.0, *p* = 0.006) and 12-month (from 87.1 to 91.0, *p* = 0.006) follow-up visits on 29 and 15 patients, respectively. This outcome was related to the improvement of some PSG parameters, specifically W (%), WASO, and Aw/h. After 3 months (*n* = 29), median W percentage was changed from 11.9 to 9.0 (*p* = 0.008), median WASO from 33.0 to 23.0 min (*p* = 0.001), and median Aw/h from 4.0 to 3.5 (*p* = 0.001) (see Table 5).

These results were confirmed at 12-month follow-up on 15 patients, particularly the reduction in median W percentage (*p* = 0.003) and WASO (*p* = 0.012); change in median Aw/h did not reach the statistical significance (*p* = 0.094), whereas median SL was significantly decreased from 16.0 to 9.0 min (*p* = 0.014) (see Table 6).

Interestingly, we found a weak positive correlation between several parameters related to migraine improvement (i.e., reduction in MMD, MIDAS score, severity, and duration of attacks) and the increase in objective SE, both after 3 and 12 months of treatment with erenumab (see Supplementary Table 2). This trend became statistically significant only for attacks severity at T1.

Sleep distribution in N1, N2, N3, and REM sleep stages was similar at baseline and at times T1 and T2. Similarly, we did not find significant changes in other PSG parameters, such as TST, RL, and Ar/h, during the follow-up (see Tables 5, 6).

## Discussion

To our knowledge, this is the first polysomnographic study that evaluated objective sleep changes in patients who were treated with MPMs. A recent meta-analysis that includes trials based on PSQI and PSG demonstrated poorer sleep quality and a lower percentage of REM sleep in patients with migraine when compared to healthy controls (9). In our patients, we found a higher SE after the treatment with erenumab. This result was obtained through a significant reduction of wake time duration, WASO, and number of awakenings per hour, with unaltered TST and duration of different sleep stages, indicating a higher sleep stability after erenumab treatment. Particularly, we observed a weak positive relationship between the improvement in objective SE and a better response to the treatment with erenumab. This association, however, did not reach a clear statistical significance probably due to the limited sample size. Subjective measures of sleep quality (PSQI and ESS questionnaires) corroborated home-PSG data. Our patients reported to sleep better after only 3 months of treatment, reaching a significant decrease in EDS and daytime dysfunction after 12 months.

Duman et al. used for the first time the PSQI questionnaire and testing the subjective effects of migraine prophylaxis on sleep (16). In that study, patients were treated with propranolol or amitriptyline for 12 weeks, after which a significant improvement in sleep quality was observed in both groups, with greater results in those with initial PSQI scores ≥6 taking amitriptyline. These molecules exert central effects on sleep architecture by reducing REM sleep. In particular, it is well-known the sleep-promoting role of amitriptyline by modulating sleep-wake cycle neurotransmitters (36–38). Erenumab, in contrast, does not cross the blood-brain barrier (BBB) (39, 40), and thus a direct action on sleep central pathways is not expected. For this reason, the observed sleep changes can be attributed to migraine improvement itself. In fact, our PSG results occurred concurrently with erenumab effectiveness in reducing migraine frequency, severity, duration of attacks and improving the response to symptomatic medications and the disability related to migraine attacks. We also observed a significant improvement in patients' mood and self-reported quality of life. In this respect, our data are consistent with the increasing amount of literature demonstrating erenumab success in migraine prevention, with concurrent benefits on living standards and mood (17, 18, 20, 41–44). Real-life data support erenumab safety and tolerability. We detected infrequent and mild AEs, obtaining full adherence to the treatment. Compared to information in the Summary of Product Characteristics (45), we found a higher frequency of constipation (20.7%); however, none of the patients dropped out by reason of side effects, and constipation tended to dissipate over time, as described elsewhere (41, 42, 46).

Erenumab was the first monoclonal antibody approved by the U.S. Food Drug Administration and the European Medicines Agency in May 2018 for the treatment of chronic and episodic migraine (45, 47). Its efficacy is mainly attributed to the blockage of CGRP receptors expressed in the trigeminal system. CGRP is a vasoactive neuropeptide released from trigeminal ganglia cells at the level of intracranial cerebral and dural blood vessels, causing vasodilation and neurogenic inflammation and playing an important role in sustaining a state of central sensitization during migraine attacks (39, 40, 48).

Migraine and sleep share a complex bidirectional relationship, which still remains poorly understood. At least in part, they have common anatomic structures, neural pathways, signaling neurotransmitters, and neuropeptides. Both the brainstem and the hypothalamus play a central role in migraine and sleep-wake cycle regulation, particularly involving dopamine, serotonin, adenosine, melatonin, orexin, and pituitary adenylate cyclase-activating polypeptide (PACAP) as possible mediators of this relationship (49–51).

Patients with migraine were found with low serotonin levels during interictal periods and high mobilization of serotonin intracellular stores in the early phase of the attack, promoting wakefulness, disrupted sleep, and REM sleep reduction (52, 53). Instead, inhibition of serotoninergic neurons of the dorsal raphe nucleus in the brainstem is essential for the transition from NREM to REM sleep (54). Dopaminergic A11 hypothalamic nucleus, implicated in the prodromal symptoms of migraine, facilitates arousals and wakefulness and could explain the high prevalence of RLS in migraine patients (51). The dopamine neurons of the periaqueductal gray are also involved in wake promotion by inhibiting the hypothalamus, and in pain signaling between the trigeminocervical complex (TCC), the hypothalamus, and the thalamus. Noradrenergic neurons in the locus coeruleus work in concert with dopaminergic and serotoninergic systems of the brainstem as pain modulators and facilitate wakefulness (50, 55–57). Moreover, a possible association between the therapeutic effect of sleep in migraine and the glymphatic system (a little-known waste removal mechanism working during sleep in the central nervous system) has been hypothesized; its dysfunction has been also proposed as a possible mechanism in the development of chronic migraine (49, 58).

Finally, PACAP is emerging as an important molecular target in the pathophysiology of migraine. Similar to CGRP, it works on the nociceptive neurons of the TCC promoting vasodilation and increased cerebral blood flow during migraine attacks (59, 60). Recent evidence suggests that PACAP also plays an important role in the hypothalamus for sleep homeostasis. In animal models, it seems to act on sleep-related neuroendocrine changes, through the modulation of sleep-wake cycle pathway, clock genes expression, and melatonin synthesis (61–65). Despite current knowledge, the pathogenic correlation between regulation of sleep, migraine, and circadian rhythm remains largely unclear. PACAP and CGRP represent significant migraine triggers, but little is known about their circadian variability. Again, nothing is known about possible indirect central effects due to peripheral modulation of CGRP with erenumab or other anti-CGRP monoclonal antibodies.

This study has several limitations. The first one is represented by the small size of the sample, due to the strict enrollment criteria, and the even smaller size of the group that completed the 12-month follow-up. Sample reduction at 12 months was determined randomly, mainly because of limited hospital access during the COVID-19 pandemic and, rather, it was not influenced by factors such as patients' sleep quality or therapeutic response to erenumab. Despite some disadvantages when compared to laboratory recordings, home-PSG was a more cost-effective alternative, easy to perform during the COVID-19 pandemic, and enabled patients to sleep in their habitual setting. Another issue is the absence of a control group in the study design, although it has to be considered that since erenumab does not pass the BBB, we studied the impact of migraine improvement on sleep, rather than the direct effect of the drug on patients' sleep. Moreover, we are aware of the fact that a subgroup of patients continued to take other MPMs with a potential influence on sleep during the treatment with erenumab (i.e., antidepressants, antihistamines, and beta-blockers). In order to prevent a possible interference of these drugs on our results, however, other MPMs were not modified during the 2 months preceding the enrollment and for the following 3 months. Interestingly, during the second study period, we confirmed a global improvement in sleep quality although antidepressants had been discontinued in several cases. This gives more strength to the positive effect of erenumab on sleep, since this effect was obtained despite the withdrawal of other sleep-promoting drugs. One last point to consider is that most of the patients selected, due to the enrollment criteria, already had a quite normal SE at baseline. This precondition explains the mild but significant extent of results, since an impressive change in sleep quality was not expected.

Future studies on larger groups will be needed in order to perform further analyses, for instance, to evaluate whether there is a linear correlation between migraine improvement and sleep changes, or whether results are influenced by the treatment dose or the type of monoclonal antibody used. Explaining common pathophysiologic mechanisms between migraine, sleep homeostasis, and sleep disorders still remains very challenging.

In conclusion, erenumab is effective and safe in migraine prevention, demonstrating consequent benefits on both subjective sleep quality and sleep architecture. After starting the treatment, our patients significantly increased their SE, resulting in better life quality, improved daytime functioning, and reduced somnolence levels. Our findings support the growing body of evidence suggesting a common undelying pathophysiology for migraine and sleep disorders. Basic research into neuropeptides and their pathways appear to be crucial to improve our understanding of this fascinating relationship and developing new therapeutic solutions.

## Data Availability Statement

The raw data supporting the conclusions of this article will be made available by the authors, without undue reservation.

## Ethics Statement

The studies involving human participants were reviewed and approved by Comitato Etico Unico Regionale, Friuli Venezia Giulia (CEUR FVG), Italy. The patients/participants provided their written informed consent to participate in this study.

## Author Contributions

GP, SP, and MV conceived the study and its design. GP, SP, and AS performed the acquisition and interpretation of data. GP and SP drafted the manuscript. AN contributed to the analysis and interpretation of data. AN, AS, GG, and MV substantively revised the manuscript. All authors made substantial contributions to conception, study design, data collection, and approved the final manuscript.

## Conflict of Interest

GP and SP received travel grants from Novartis and Eli Lilly. AS received travel grants from Novartis. MV received travel grants, honoraria for scientific contributions or clinical investigation studies from Novartis, Teva Pharmaceuticals, and Eli Lilly. The remaining authors declare that the research was conducted in the absence of any commercial or financial relationships that could be construed as a potential conflict of interest.

## Publisher's Note

All claims expressed in this article are solely those of the authors and do not necessarily represent those of their affiliated organizations, or those of the publisher, the editors and the reviewers. Any product that may be evaluated in this article, or claim that may be made by its manufacturer, is not guaranteed or endorsed by the publisher.

## Abbreviations

ICHD-III: International Classification of Headache Disorders, 3rd edition
EDS: excessive daytime sleepiness
SE: sleep efficiency
RLS: restless legs syndrome
MPM: migraine-preventive medication
CGRP: calcitonin gene-related peptide
PSG: polysomnography
MMD: monthly migraine days
PSQI: Pittsburgh Sleep Quality Index
ESS: Epworth Sleepiness Scale
MIDAS: Migraine Impact and Disability Assessment Scale
HIT-6: Headache Impact Test, 6th edition
BDI-II: Beck Depression Inventory-II
EQ-VAS: EuroQoL Visual Analogue Scale
MOH: medication overuse headache
BoNT-A: botulinum neurotoxin type A
TST: total sleep time
REM: rapid eye movement
WASO: wake after sleep onset
SL: sleep latency
RL: REM latency
AE: adverse event
BBB: blood-brain barrier
PACAP: pituitary adenylate cyclase-activating polypeptide
TCC: trigeminocervical complex.
