# Supplementary material for: Erenumab Impact on Sleep Assessed With Questionnaires and Home-Polysomnography in Patients With Migraine: The ERESON Study
Source: Front Neurol. 2022 May 13;13:869677. doi: 10.3389/fneur.2022.869677 (PMC9136084; doi:10.3389/fneur.2022.869677)
Supplement: Supplementary file 1 [file Data_Sheet_1.docx]

Supplementary Material

**Supplementary Table 1. Sleep profiler^TM^ specifics and supplementary information on acquisition and analysis of polysomnographic signals.**

| Signals | EEG, EOG, EMG |
| --- | --- |
| EEG derivations | AF7-AF8, AF7-Fpz, AF8-Fpz |
| Pulse rate | Forehead photoplethesmography |
| Snoring | Integrated acoustic microphone |
| Head position/movement | Triaxial accelerometer (converting X/Y/Z signals to 360-degree angles) |
| Patient training | Skin cleansing, device self-positioning, device vocal instructions, record starting/stopping, electrodes replacing |
| Software signal filters | 0.75 Hz high-pass filter, band-stop filter (to attenuate sweat artifacts), 16 Hz band-pass filter |
| Automated scoring system | Based on sleep markers on each 30-second epoch |
| Sleep markers | Sleep spindles (brief bursts in alpha-sigma power), cortical arousals (increased alpha power for >3 seconds), microarousals as a (combination of increased alpha and/or EMG power for > 3 seconds). SEM and REM (distinguished by AF7-FPz and AF8-FPz signal patterns) |

EEG = electroencephalography, EOG = electrooculography, EMG = electromyography, SEM = slow eye movements, REM = rapid eye movements.

**Supplementary Table 2. Results of comparisons between change in objective sleep efficiency and variables associated with migraine response to erenumab.**

| **Comparisons after three months of erenumab treatment (n = 29)** ***^†^*** | | | |
| --- | --- | --- | --- |
|  | SE < 90% | SE ≥ 90% |  |
| MMD < 50% reduction, n (%) | 10 (34.5) | 11 (38.0) | p = 0.250 |
| MMD ≥ 50% reduction, n (%) | 2 (6.9) | 6 (20.6) |  |
| MIDAS score < 50% reduction, n (%) | 8 (27.6) | 9 (31.0) | p = 0.363 |
| MIDAS score ≥ 50% reduction, n (%) | 4 (13.8) | 8 (27.6) |  |
| No improved attack severity, n (%) | 12 (41.4) | 3 (10.4) | p = 0.020* |
| Improved attack severity, n (%) | 5 (17.2) | 9 (31.0) |  |
| No subjective reduction in migraine duration, n (%) | 4 (13.8) | 5 (17.2) | p = 0.568 |
| Subjective reduction in migraine duration, n (%) | 8 (27.6) | 12 (41.4) |  |
| **Comparisons after 12 months of erenumab treatment (n = 15) *^†^*** | | | |
|  | SE < 90% | SE ≥ 90% |  |
| MMD < 50% reduction, n (%) | 3 (20.0) | 4 (26.7) | p = 0.427 |
| MMD ≥ 50% reduction, n (%) | 2 (13.3) | 6 (40.0) |  |
| MIDAS score < 50% reduction, n (%) | 1 (6.7) | 1 (6.7) | p = 0.571 |
| MIDAS score ≥ 50% reduction, n (%) | 4 (26.7) | 9 (60.0) |  |
| No improved attack severity, n (%) | 1 (6.7) | 2 (13.3) | p = 0.758 |
| Improved attack severity, n (%) | 4 (26.7) | 8 (53.3) |  |
| No subjective reduction in migraine duration, n (%) | 3 (20.0) | 1 (6.7) | p = 0.077 |
| Subjective reduction in migraine duration, n (%) | 2 (13.3) | 9 (60.0) |  |

n = number, SE = objective sleep efficiency; MMD = monthly migraine days; MIDAS = Migraine Impact and Disability Assessment Scale. ***†*** *Fisher’s exact test was used for comparisons.*

**Supplementary figure 1. Depiction of Sleep Profiler™**

**
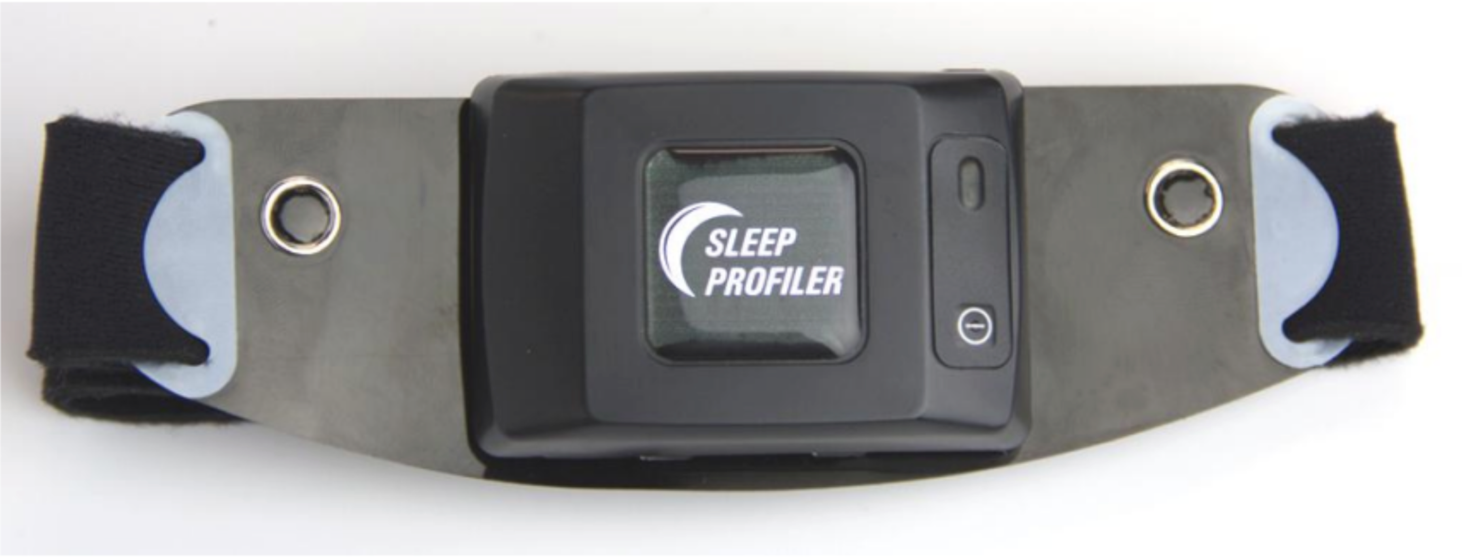
**

**Supplementary figure 2. Presentation of a 30-second epoch of NREM2 sleep stage of a patient included in the study.**

**
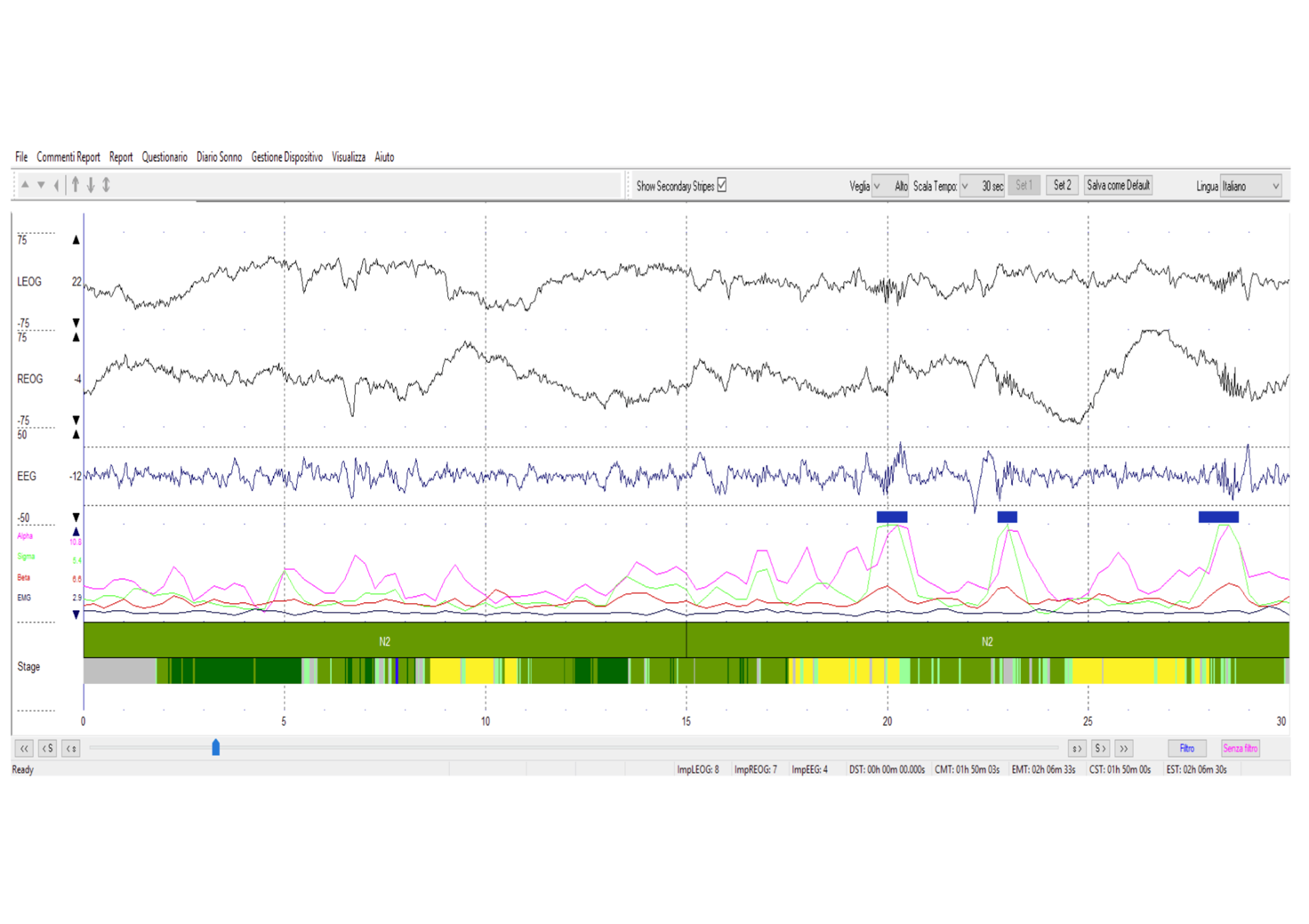
**Sleep Profiler™ signals including EEG (LEOG, REOG, and EEG), power spectrum (Alpha, Sigma, Beta, EMG are automatically viewed; Delta and Theta must be turned on to be viewed), and two stripes displaying the sleep stage and hypnogram. Three blue stripes below the EEG signal identify sleep spindles. EEG = electroencephalography, LEOG = left electrooculography, REOG = right electrooculography, EMG = electromyography.

**Supplementary figure 3. Extract from a Sleep Profiler^TM^ polysomnography report of a patient included in the study.**


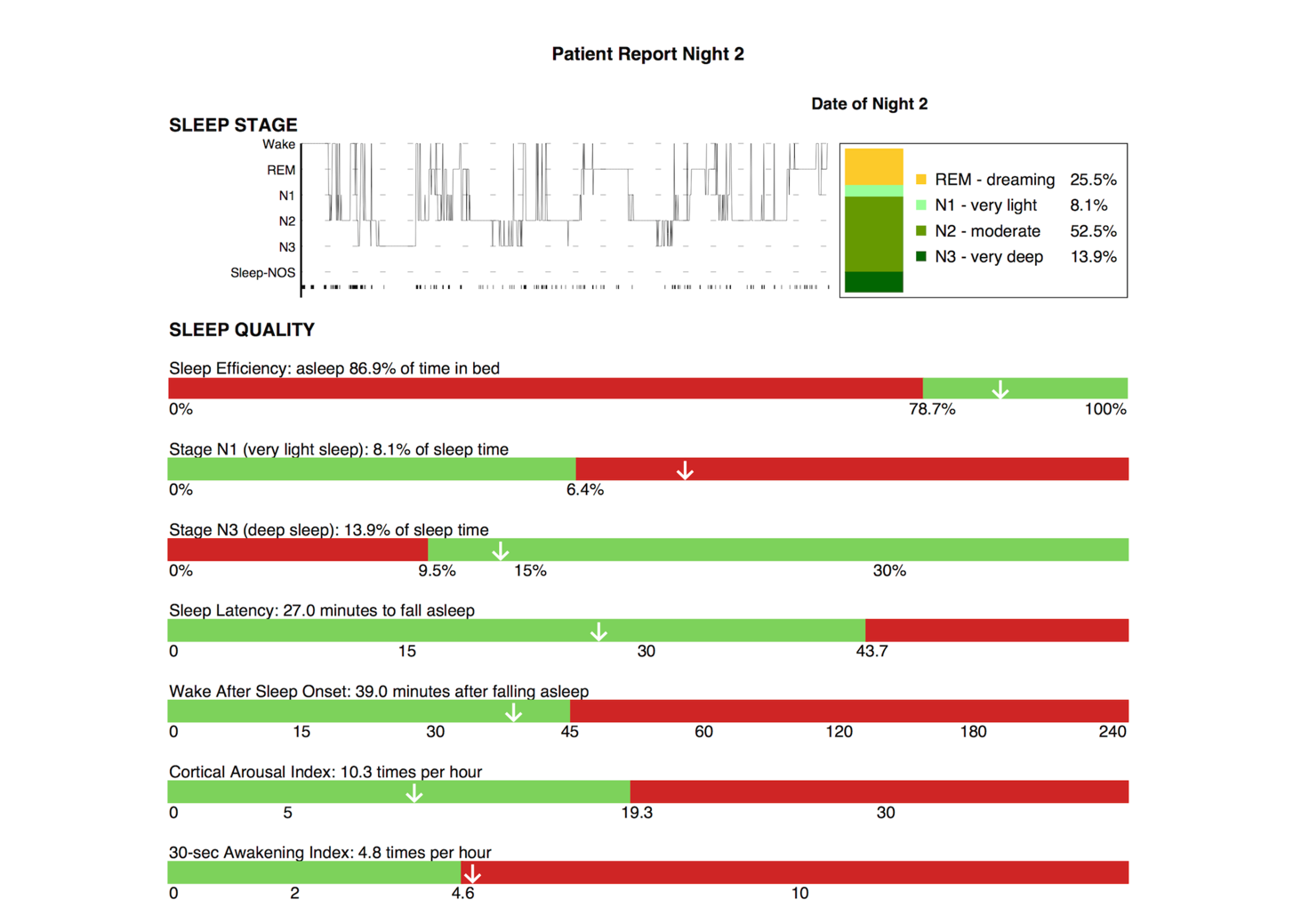


Sleep Profiler™ Report includes hypnogram, percentage of different sleep stage and parameters to evaluate sleep quality. N1 = NREM1 sleep stage; N2 = NREM2 sleep stage; N3 = NREM3 sleep stage; REM = REM (Rapid Eye Movement) sleep stage.
